# Synthesis and Characterization of Conducting PANDB/χ-Al_2_O_3_ Core-Shell Nanocomposites by In Situ Polymerization

**DOI:** 10.3390/polym13162787

**Published:** 2021-08-19

**Authors:** Cheng-Ho Chen, Ying-Chen Lin, Fu-Su Yen

**Affiliations:** 1 Department of Chemical and Materials Engineering, Southern Taiwan University of Science and Technology, Tainan City 710, Taiwan; ma340101@stust.edu.tw; 2 Department of Resources Engineering, National Cheng-Kung University, Tainan City 701, Taiwan; yfs42041@mail.ncku.edu.tw

**Keywords:** polyaniline, dodecylbenzenesulfonic acid, χ-Al_2_O_3_, in situ polymerization, core-shell nanocomposite

## Abstract

Polyaniline doped with dodecylbenzenesulfonic acid/χ-aluminum oxide (PANDB/χ-Al_2_O_3_) conducting core-shell nanocomposites was synthesized via an in situ polymerization method in this study. PANDB was synthesized in the presence of dodecylbenzenesulfonic acid (DBSA), which functioned as a dopant and surfactant. The electrical conductivity of the conducting PANDB/χ-Al_2_O_3_ core-shell nanocomposite was approximately 1.7 × 10^−1^ S/cm when the aniline/χ-Al_2_O_3_ (AN/χ-Al_2_O_3_) weight ratio was 1.5. The transmission electron microscopy (TEM) results indicated that the χ-Al_2_O_3_ nanoflakes were thoroughly coated by PANDB to form the core-shell (χ-Al_2_O_3_-PANDB) structure. The TEM and field-emission scanning electron microscopy (FE-SEM) images of the conducting PANDB/χ-Al_2_O_3_ core-shell nanocomposites also indicated that the thickness of the PANDB layer (shell) could be increased as the weight ratio of AN/χ-Al_2_O_3_ was increased. In this study, the optimum weight ratio of AN/χ-Al_2_O_3_ was identified as 1.5. The conducting PANDB/χ-Al_2_O_3_ core-shell nanocomposite was then blended with water-based polyurethane (WPU) to form a conducting WPU/PANDB/χ-Al_2_O_3_ blend film. The resulting blend film has promising antistatic and electrostatic discharge (ESD) properties.

## 1. Introduction

Polyaniline (PANI) is an important member of the intrinsically conducting polymer (ICP) family. Because of its unique electrochemical properties and environmental stability, PANI has been extensively studied by numerous researchers [1,2,3,4,5,6] and has been employed in many applications, such as secondary batteries [7,8], biosensors [9,10], corrosion protectors [11,12], antistatic packaging materials [13], and light-emitting diodes (LEDs) [14].

In recent years, encapsulating inorganic materials inside a PANI shell has become the most popular and interesting aspect of composites. The combination of inorganic components with conducting PANI has attracted considerable attention because of the resulting composites’ novel physical and chemical properties and potential applications. These core-shell conducting composites can provide new synergistic properties that cannot be attained from the individual materials alone.

Preparing conducting core-shell PANI/inorganic composites via conventional mixing or blending in melt or solution form is difficult because PANI is insoluble, infusible, and possesses poor mechanical properties. One of the most promising methods to prepare conducting core-shell PANI/inorganic composites is through an in situ chemical polymerization of a monomer in the presence of inorganic components. Many groups have reported conducting PANI/inorganic core-shell composites such as PANI/Y_2_O_3_ [15], PANI/Zn_x_Fe_3−x_O_4_ [16], PANI/T-ZnOw [17], PANI/TiO_2_ [18], PANI/MnO_2_ [19], PANI/ZrO_2_ [20], PANI/alumina [21,22,23], PANI/attapulgite (ATP) [24], and PANI/clay [25].

However, PANI doped with strong protonic acids is intractable. There have been a number of attempts towards making conducting PANI processable by doping with various dopants. The most attractive and promising system is PANI doped with dodecylbenzenesulfonic acid (DBSA) (PANDB). This is because DBSA containing a polar head and a long nonpolar chain which can function both as a surfactant and dopant. This advantage allows PANDB to not only become soluble in various organic solvents (e.g., chloroform and xylene) but also to have better compatibility with hydrophobic polymers. Therefore, conducting PANI doped with DBSA (PANDB) was adopted in this study.

The χ-Al_2_O_3_ nanoflake is one of the metastable polymorphs of transition alumina with a relatively high aspect ratio, thermal stability, porosity, and specific surface area. Due to its flake structure and thickness of around 20~50 nm, the χ-Al_2_O_3_ nanoflake can be used as an excellent reinforcement to enhance both the strength and stiffness of semicrystalline and glassy thermoplastics, elastomers, and epoxy resins.

To the best of our knowledge, no studies have reported the preparation for conducting PANDB/χ-Al_2_O_3_ core-shell nanocomposites through an in situ polymerization process in the presence of DBSA as the dopant. The goal of this study is to improve the electrical conductivity of the χ-Al_2_O_3_ nanoflake and let the χ-Al_2_O_3_ nanoflake coated with conducting PANDB form the conducting PANDB/χ-Al_2_O_3_ core-shell nanocomposites. In addition, the optimum weight ratio of aniline/χ-Al_2_O_3_ (AN/χ-Al_2_O_3_) to synthesize the conducting PANDB/χ-Al_2_O_3_ core-shell nanocomposites is also determined. Moreover, we also discuss the influences of the weight ratio of AN/χ-Al_2_O_3_ on the electrical conductivity, chemical structure, and morphology of the conducting PANDB/χ-Al_2_O_3_ core-shell nanocomposites. Finally, the synthesized product will be blended with water-based polyurethane (WPU) to form a conducting WPU/PANDB/χ-Al_2_O_3_ blend film. The antistatic tests of the surface of the pure WPU film and the conducting WPU/PANDB/χ-Al_2_O_3_ blend film will be examined and compared.

## 2. Experimental Section

### 2.1. Materials

Aniline (AN) and ammonium persulfate (APS) were purchased from Merck KGaA (Darmstadt, Germany). Dodecylbenzene sulfonic acid (DBSA) was purchased from Tokyo Kasei Kogyo Co., Ltd. (Tokyo, Japan). The porous χ-Al_2_O_3_ nanoflake powder, which has a high porosity and high specific surface area, was supplied by the Particulate Research Center at the National Cheng-Kung University, Tainan City, Taiwan. The sectional diameter of one χ-Al_2_O_3_ nanoflake was in the range of 150 to 500 μm, and the thickness of one χ-Al_2_O_3_ nanoflake was in the range of 20 to 50 nm. Meanwhile, the specific surface area of the χ-Al_2_O_3_ nanoflake was in the range of 130 to 150 m^2^/g. The nonionic WPU dispersion, tackiness, and curing agents for WPU were supplied by Hailsun Chemical Co., Ltd. (Taipei City, Taiwan).

### 2.2. Synthesis of Pure PANDB and Conducting PANDB/χ-Al_2_O_3_ Core-Shell Nanocomposites

Pure PANDB was directly prepared by a chemical oxidation polymerization according to the modified procedure described in our previous study [26,27]. 8 g of aniline was mixed with 23 g of DBSA and 400 mL of distilled water to form a homogeneous milky white dispersion of anilinium-DBSA complex in a 1000 mL four-necked flat-bottom reactor under proper stirring at room temperature. The APS solution (20 g of APS dissolved in 200 mL of distilled water) was then slowly added to the reactor. The aniline:DBSA:APS molar ratio was set as 1:0.8:1. After a 2 h reaction, the darkish-green PANDB dispersion was precipitated by the addition of 600 mL of acetone. The reaction mixture was then filtered and washed several times with deionized water until the filtrate was colorless. Finally, the product was collected and dried in an oven at 60 °C for 24 h.

**Scheme 1:**
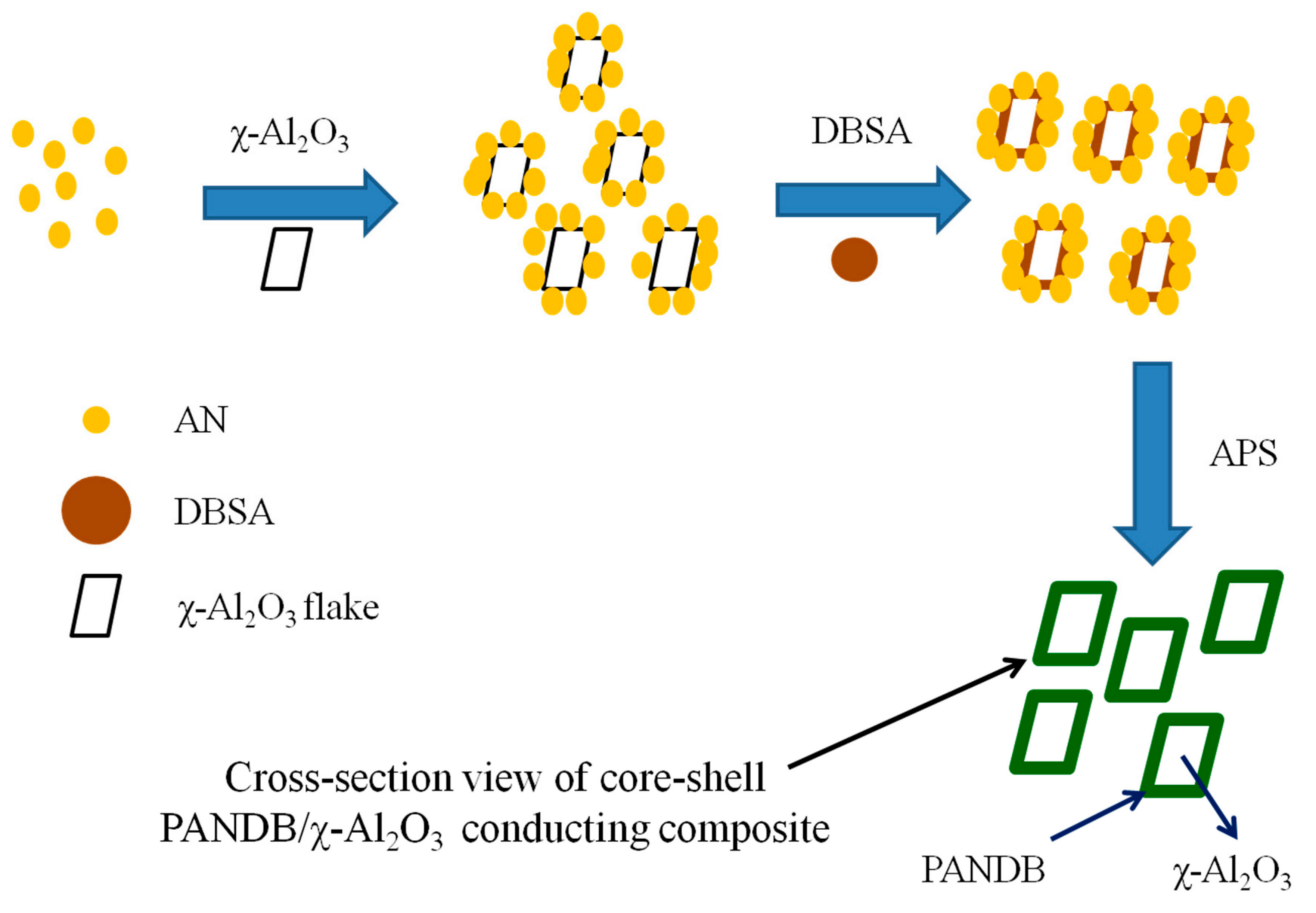
Synthesis process of the conducting PANDB/χ-Al_2_O_3_ core-shell nanocomposite via an in situ polymerization.

The conducting PANDB/χ-Al_2_O_3_ core-shell nanocomposite was synthesized via an in situ chemical oxidation polymerization. 2 g of χ-Al_2_O_3_ was dispersed in 100 mL of an aqueous solution that contained 1 g of aniline. The resulting dispersion was then stirred at room temperature for 10 min. The AN/χ-Al_2_O_3_ weight ratio was set as 0.5. Then, 50 mL of an aqueous DBSA solution was added to the solution. After 10 min, 30 mL of an aqueous APS solution was added dropwise to the dispersion with constant stirring. The aniline:DBSA:APS molar ratio was 1: 0.8: 1. The resulting mixture was allowed to react for 2 h at room temperature. Subsequently, the product was washed with deionized water until the filtrate became colorless; the product was then dried in a vacuum oven at 60 °C for 24 h. To determine the effect of the weight ratio of AN/χ-Al_2_O_3_ on the properties of the conducting PANDB/χ-Al_2_O_3_ core-shell nanocomposites, products with weight ratios of 1.0, 1.5, and 2.0 were also prepared. Scheme 1 shows the synthesis of the conducting PANDB/χ-Al_2_O_3_ core-shell nanocomposite via an in situ polymerization process.

### 2.3. Fabrication of Conducting WPU/PANDB/χ-Al_2_O_3_ Blend Film

WPU dispersion was placed into a beaker equipped with a mechanical stirrer. 20wt% of the synthesized conducting PANDB/χ-Al_2_O_3_ core-shell nanocomposite was added to the WPU dispersion. The WPU/20wt% (PANDB/χ-Al_2_O_3_) mixture was then thoroughly mixed. Next, a tackiness agent was added to the mixture under mechanical stirring until the mixture viscosity reached around 7500 cps. A curing agent of 1.5 g was added to the mixture sequentially and was completely and well mixed. The resulting mixture was degassed with a vacuum pump to eliminate air bubbles. Finally, the air bubble-free mixture was cast on a polyethylene terephthalate (PET) substrate and heated to 120 °C for 2 min, then 150 °C for 2 min to complete the curing reaction of WPU. For a comparison, the pure WPU film was also made.

### 2.4. Characterization

#### 2.4.1. Electrical Conductivity and Surface Resistance Analysis

In total, 0.1 g of sample was weighed and then pressed under 3.0 × 10^5^ psi for 2 min at room temperature. The electrical conductivity σ (S/cm) of the sample was examined by a four-point probe meter (model: LSR4-KHT200, KeithLink Technology Co., Ltd., Taipei City, Taiwan) at room temperature. Each sample was first compressed into a sample with a diameter of 10 mm. Then, 10 different locations on the sample surface were measured and averaged. The obtained results were averaged and reported. The surface resistance (Ω/sq) of the pure WPU film and conducting WPU/20wt%(PANDB/χ-Al_2_O_3_) blend films was examined by the same equipment. 10 different locations on the sample surface were measured and averaged.

#### 2.4.2. FTIR Analysis

The chemical structure of the sample was determined by Fourier-transform infrared spectroscopy (FTIR) (model Spectrum One; Perkin Elmer, Waltham, MA, USA) over the wavenumber range 400–4000 cm^−1^ at 32 scan/s. The powdered samples and potassium bromide (KBr) (weight ratio of approximately 1:99) were grounded together into fine powders, and the homogeneous mixture was then pressed into a pellet for analysis.

#### 2.4.3. UV-Vis Analysis

The sample was dispersed in absolute alcohol under ultrasonic agitation for 1 h at room temperature. An ultraviolet-visible spectrophotometer (UV-Vis; model UV-2401 PC, Shimadzu Co., Ltd., Kyoto, Japan) was used to measure the optical absorbance of the sample solution at a wavelength range of 300 to 900 nm.

#### 2.4.4. XRD Analysis

X-ray diffraction (XRD) patterns of the synthesized sample were obtained at room temperature using an X-ray diffractometer (Rigaku MultiFlex ZD3609N, Rigaku Co., Tokyo, Japan) equipped with a Cu-Kα radiation source (λ = 0.154 nm). The diffraction patterns were recorded at a scan rate of 2°/min over the 2θ range from 5° to 80°.

#### 2.4.5. TEM Examination

The sample was examined by transmission electron microscopy (TEM, model JEM-1230, JEOL, Ltd., Tokyo, Japan) by casting a diluted dispersion of the sample onto a carbon-coated copper grid. The TEM was operated at an accelerating voltage of 80 kV.

#### 2.4.6. FE-SEM Examination

The sample was dried after filtration. The sample was then fractured, and each sample was coated with a gold-palladium film. The surface morphologies of the sample were examined using a field-emission scanning electron microscope (FE-SEM, model JSM 6700F; JEOL, Ltd., Tokyo, Japan).

## 3. Results and Discussion

Table 1 shows the electrical conductivities of the pure PANDB and the conducting PANDB/χ-Al_2_O_3_ core-shell nanocomposites. The electrical conductivity of the pure PANDB is approximately 6.3 × 10^−1^ S/cm. Table 1 indicates that the electrical conductivity of the conducting PANDB/χ-Al_2_O_3_ core-shell nanocomposites is increased as the weight ratio of AN/χ-Al_2_O_3_ is increased. When the weight ratio of AN/χ-Al_2_O_3_ is equal to 1.5, the conductivity of the conducting PANDB/χ-Al_2_O_3_ core-shell nanocomposite is approximately 1.7 × 10^−1^ S/cm. This result implies that when the weight ratio of AN/χ-Al_2_O_3_ is higher, the amount of PANDB generated is higher. Therefore, the surface of χ-Al_2_O_3_ can be covered more completely. As a result, the conducting path of the product increases, and the electrical conductivity increases. However, when the weight ratio of AN/χ-Al_2_O_3_ is increased to 2.0, the conductivity of the conducting PANDB/χ-Al_2_O_3_ core-shell nanocomposite remains at approximately 1.7 × 10^−1^ S/cm. This result indicates that the optimum weight ratio of AN/χ-Al_2_O_3_ is equal to 1.5 in this study.

**Figure 1:**
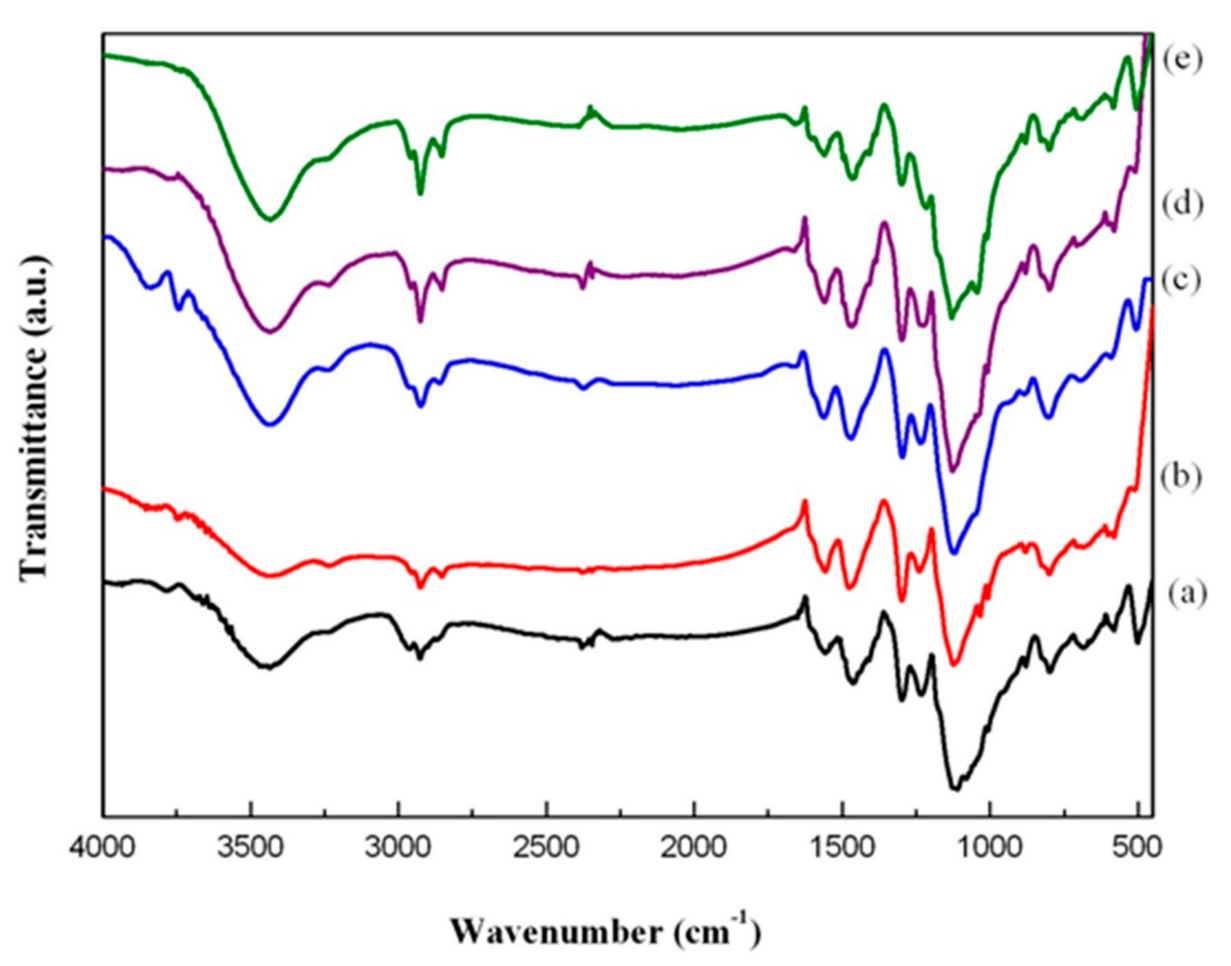
FTIR spectra of (a) pure PANDB and PANDB/χ-Al_2_O_3_ composites synthesized at AN/χ-Al_2_O_3_ weight ratios of (b) 0.5, (c) 1, (d) 1.5, and (e) 2.0.

Figure 1 shows the FTIR spectra of the pure PANDB and the conducting PANDB/χ-Al_2_O_3_ core-shell nanocomposites. The absorption peak of the −CH_2_− stretching vibration (resulting from the DBSA molecules) was observed at 2926 cm^−1^ for all of the samples. For the pure PANDB (Figure 1(a)), the characteristic peaks at 1561 and 1467 cm^−1^ are due to the stretching vibrations of the N=Q=N ring and the N–B–N ring, respectively. The characteristic peak at 1300 cm^−1^ is attributed to the C–N stretching vibration of the secondary amine in the PANDB main chain. The peaks at 998–1040 cm^−1^ are due to the asymmetric and symmetric O=S=O stretching vibrations of DBSA. The characteristic peaks at 1100–1200 cm^−1^ are due to the B–NH–Q bond or the B–NH–B bond and the in-plane bending vibration of benzenoid or quinonoid C–H (where B represents benzoic-type rings and Q represents quinonic-type rings). Because these absorption peaks were all located in the range of 1000–1200 cm^−1^, a broad peak was observed instead of each single peak in the FTIR spectrum. The peaks at 700–800 cm^−1^ are attributed to a characteristic feature of the B–NH–Q bond or the B–NH–B bond and to the out-of-plane bending vibration of benzenoid or quinonoid –C–H and –N–H bonds. Additionally, the peaks at 2900–2950 and 3267 cm^−1^ are due to the stretching vibration mode of the –C–H (–CH_3_ or –CH_2_–) and –N–H bonds, respectively. These characteristic peaks are in good agreement with the data reported in the literature [28,29].

For the conducting PANDB/χ-Al_2_O_3_ core-shell nanocomposites (Figure 1(b)–(e)), as the weight ratio of AN/χ-Al_2_O_3_ is increased, the peak at 1467 cm^−1^, which is attributed to the N–B–N ring stretching vibration mode, is slightly shifted to 1472 cm^−1^. This slight shift is due to the interaction between PANDB and the surface of the χ-Al_2_O_3_ nanoflakes. The other characteristic peaks of the conducting PANDB/χ-Al_2_O_3_ core-shell nanocomposites are similar to those of pure PANDB.

**Figure 2:**
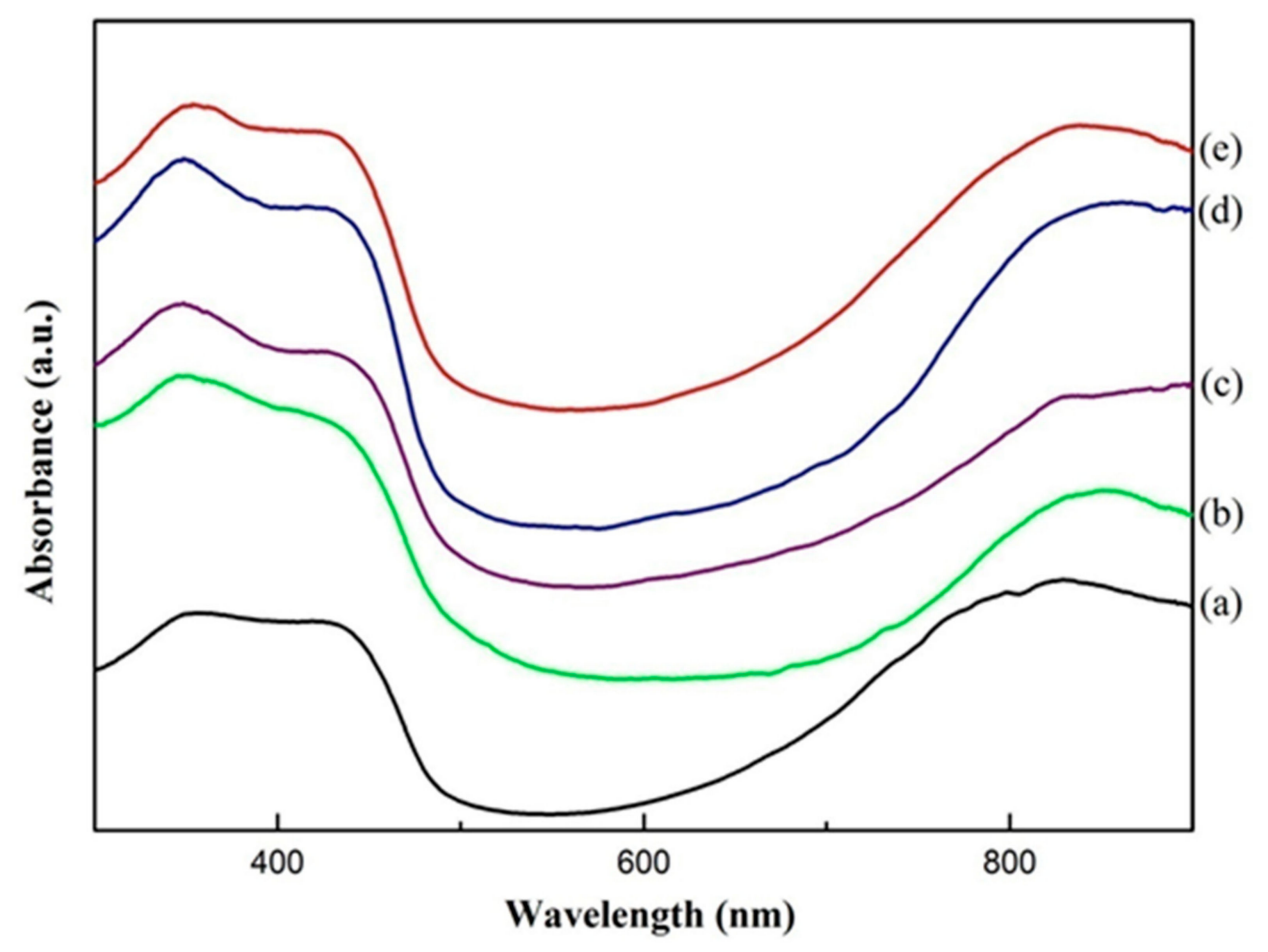
UV-Vis spectra of (a) pure PANDB and conductive PANDB/χ-Al_2_O_3_ nanocomposites synthesized at AN/χ-Al_2_O_3_ weight ratios of (b) 0.5, (c) 1, (d) 1.5, and (e) 2.0.

Figure 2 presents the UV–Vis absorption spectra of the pure PANDB and the conducting PANDB/χ-Al_2_O_3_ core-shell nanocomposites. Three characteristic absorption peaks are clearly observed in the UV–Vis spectrum of pure PANDB at approximately 350, 440, and 830 nm (Figure 2(a)). The absorption peak at approximately 350 nm is attributed to the *π*–π* transition of the benzenoid rings, whereas the peaks at approximately 440 and 830 nm are attributed to the polaron–π* transition and *π*–polaron transition, respectively [30]. The results indicate that the synthesized PANDB is an emeraldine salt (ES) PANI. Three characteristic absorption peaks also appear in the spectrum of the conducting PANDB/χ-Al_2_O_3_ core-shell nanocomposite because of the presence of PANDB (Figure 2(b)–(e)). Moreover, the intensity of the characteristic absorption peaks of the spectrum of the conducting PANDB/χ-Al_2_O_3_ core-shell nanocomposite is increased as the weight ratio of AN/χ-Al_2_O_3_ is increased. This result is attributed to more PANDB being synthesized and coated onto the surface of the χ-Al_2_O_3_ nanoflakes.

**Figure 3:**
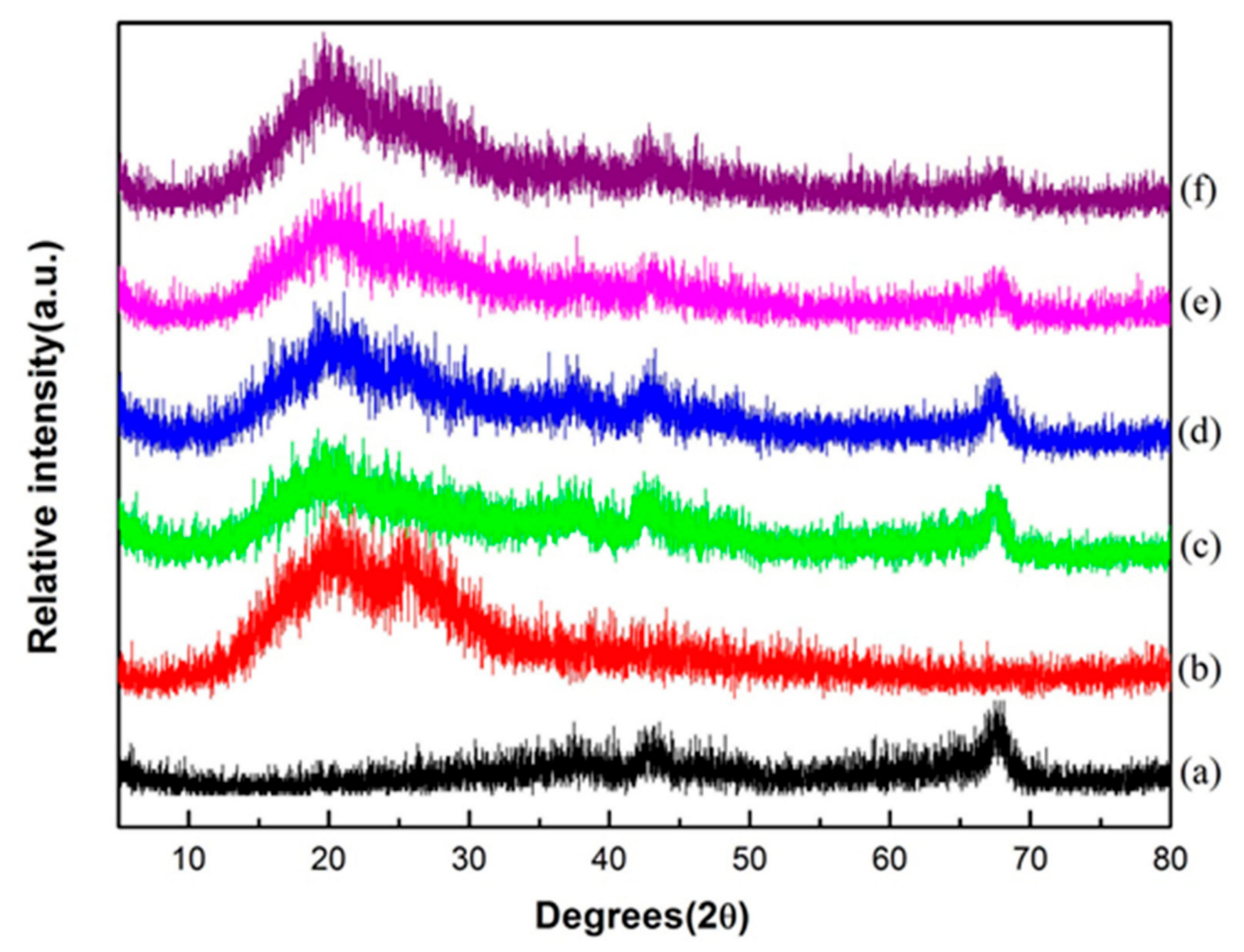
XRD patterns of (a) χ-Al_2_O_3_, (b) pure PANDB, and PANDB/χ-Al_2_O_3_ composites synthesized at AN/χ-Al_2_O_3_ weight ratios of (c) 0.5, (d) 1, (e) 1.5, and (f) 2.0.

Figure 3 presents the XRD patterns of the χ-Al_2_O_3_ nanoflakes (Figure 3(a)), pure PANDB (Figure 3(b)), and the conducting PANDB/χ-Al_2_O_3_ core-shell nanocomposites (Figure 3(c)–(f)). The pattern of the χ-Al_2_O_3_ nanoflake shows three characteristic peaks centered at 2θ = 37.4°, 43.1°, and 67.6°. The spectrum of the pure PANDB shows two characteristic peaks centered at 2θ = 20.0° and 25.8°, which are attributed to the periodicity parallel and perpendicular to the polymer chain of PANDB, respectively [31]. It can be found that the patterns of the conducting PANDB/χ-Al_2_O_3_ core-shell nanocomposites contain not only the two broad peaks centered at 2θ = 20° and 25.8° but also all of the peaks associated with the χ-Al_2_O_3_ nanoflake, which indicate that the χ-Al_2_O_3_ nanoflakes are coated by the PANDB. It can also be found that when the weight ratio of AN/χ-Al_2_O_3_ increases, the characteristic peaks of PANDB are more obvious (peaks 20.0° and 25.8°), while the characteristic peaks of χ-Al_2_O_3_ decrease (peaks 37.4°, 43.1°, and 67.6°). This is because when the weight ratio of AN/χ-Al_2_O_3_ increases, more PANDB is synthesized and there is more PAND coating on the surface of the χ-Al_2_O_3_ nanoflake.

**Figure 4:**
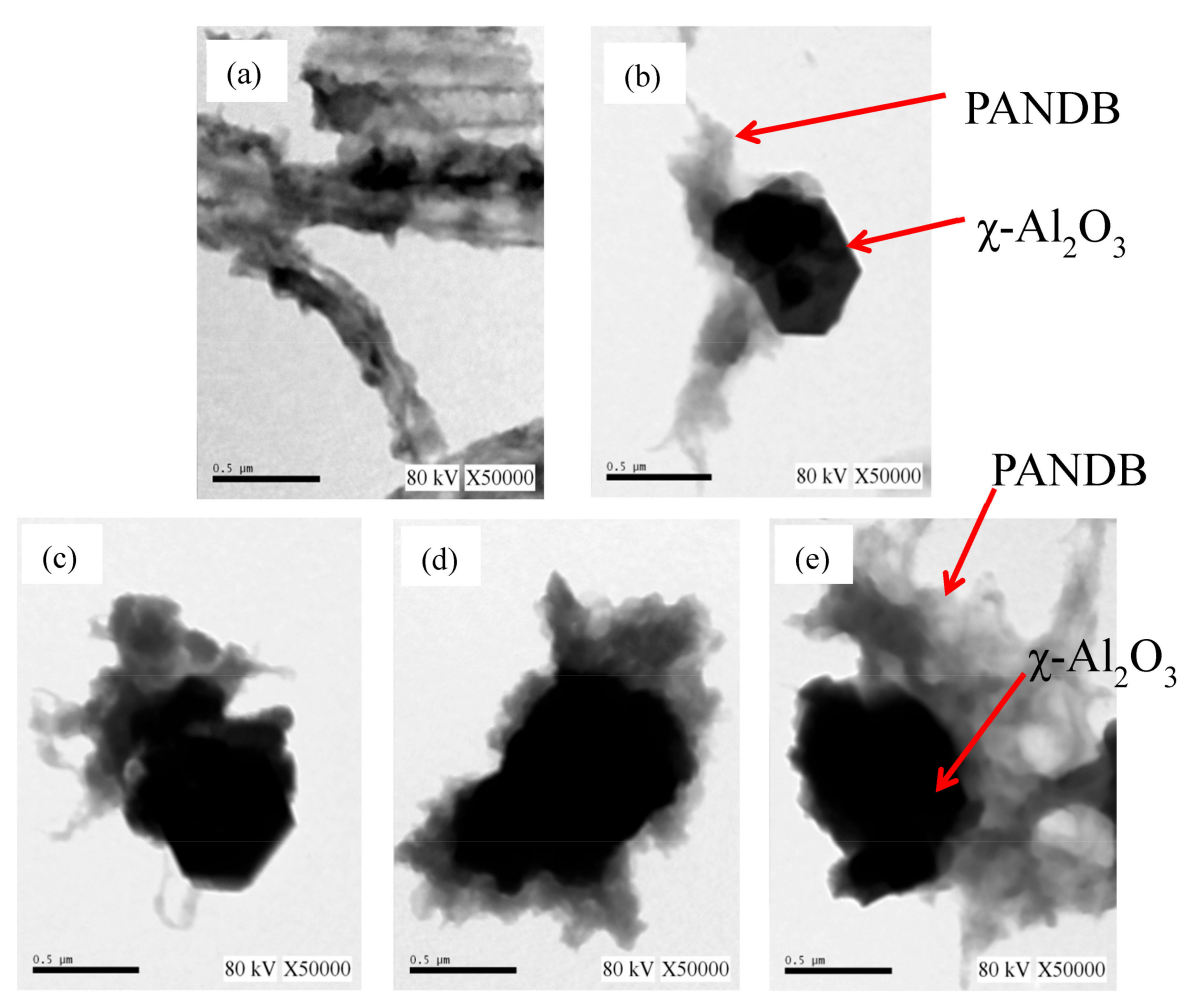
TEM images of (**a**) pure PANDB and conducting PANDB/χ-Al_2_O_3_ core-shell nanocomposites synthesized at AN/χ-Al_2_O_3_ weight ratios of (**b**) 0.5, (**c**) 1, (**d**) 1.5, and (**e**) 2.0.

Figure 4 shows the TEM images of pure PANDB and the conducting PANDB/χ-Al_2_O_3_ core-shell nanocomposites. The morphology of the pure PANDB is fiber-like. Its diameter is approximately 0.2 μm, and its length is approximately 1–3 μm (Figure 4a). For the conducting PANDB/χ-Al_2_O_3_ core-shell nanocomposites (Figure 4b–e), it can be found that the surface of the χ-Al_2_O_3_ nanoflake is coated with more PANDB as the weight ratio of AN/χ-Al_2_O_3_ is increased from 0.5 to 2.0. Meanwhile, the electrical conductivity of the PANDB/χ-Al_2_O_3_ core-shell nanocomposite is also increased from 0.6 × 10^−1^ S/cm to 1.7 × 10^−1^ S/cm (Table 1). If the weight ratio of AN/χ-Al_2_O_3_ is 1.5 or 2.0, the synthesized products have a similar electrical conductivity at approximately 1.7 × 10^−1^ S/cm. This result indicates that the surface of the χ-Al_2_O_3_ nanoflake can be completely coated when the weight ratio of AN/χ-Al_2_O_3_ is higher than 1.5 in this study. When the weight ratio of AN/χ-Al_2_O_3_ is increased, more PANDB is synthesized, and then the more conductive paths are generated.

**Figure 5:**
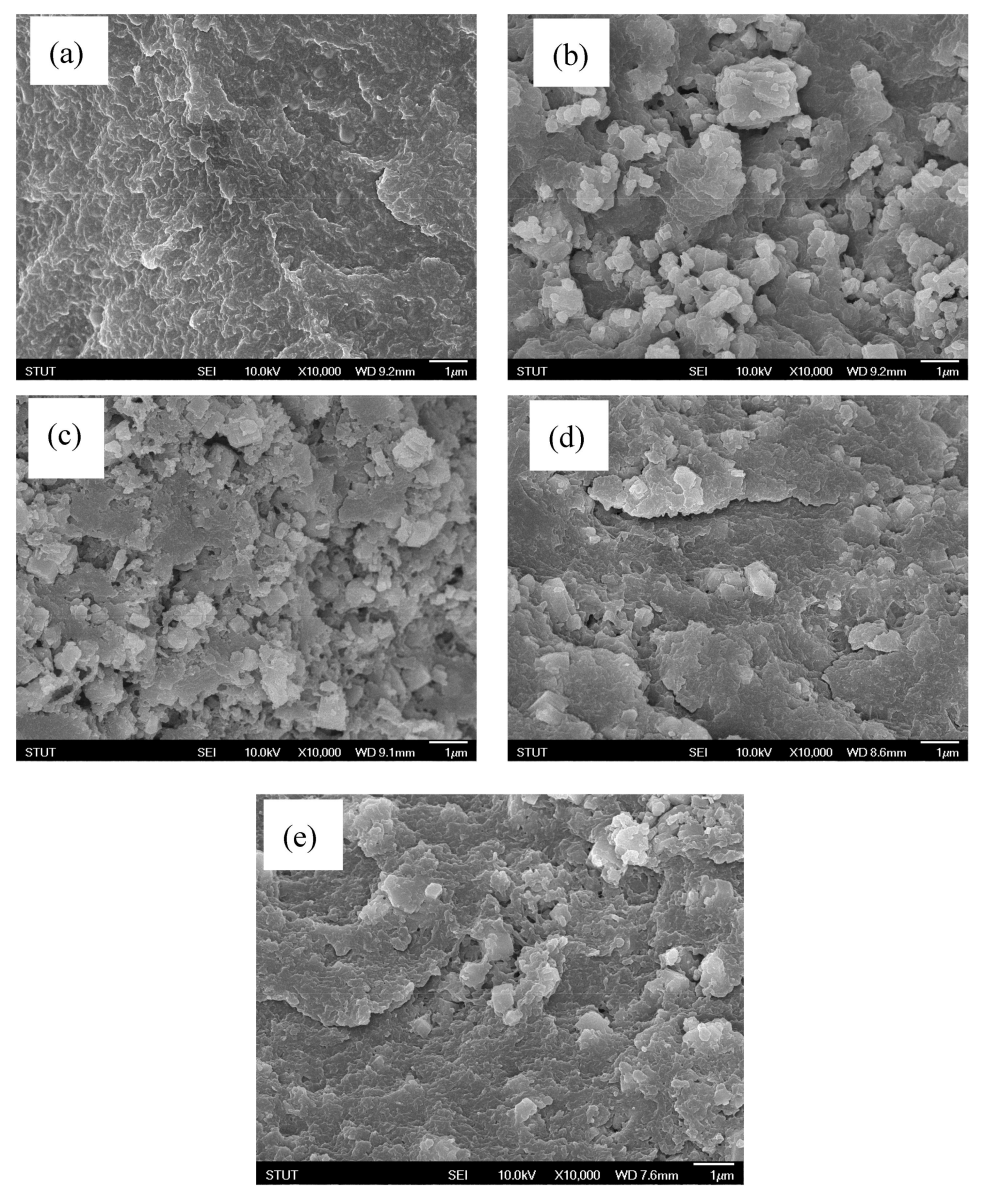
FE-SEM images of (**a**) pure PANDB and PANDB/χ-Al_2_O_3_ composites synthesized at AN/χ-Al_2_O_3_ weight ratios of (**b**) 0.5, (**c**) 1, (**d**) 1.5, and (**e**) 2.0.

Figure 5 shows the FE-SEM images of pure PANDB (Figure 5a) and the conducting PANDB/χ-Al_2_O_3_ core-shell nanocomposites (Figure 5b–d). The surface of pure PANDB exhibits a dense morphology. For the conducting PANDB/χ-Al_2_O_3_ core-shell nanocomposites, the χ-Al_2_O_3_ flakes were more thoroughly coated by PANDB when increasing the weight ratio of AN/χ-Al_2_O_3_. The surface morphologies of the conducting PANDB/χ-Al_2_O_3_ core-shell nanocomposite synthesized with AN/χ-Al_2_O_3_ weight ratios of 1.5 and 2.0 (Figure 5d,e) are similar to that of pure PANDB. This means that when the AN/χ-Al_2_O_3_ weight ratio is equal to 1.5, PANDB can cover χ-Al_2_O_3_ completely and form the conducting core-shell nanocomposite. Therefore, the optimum weight ratio of AN/χ-Al_2_O_3_ is identified as 1.5 in this study.

**Figure 6:**
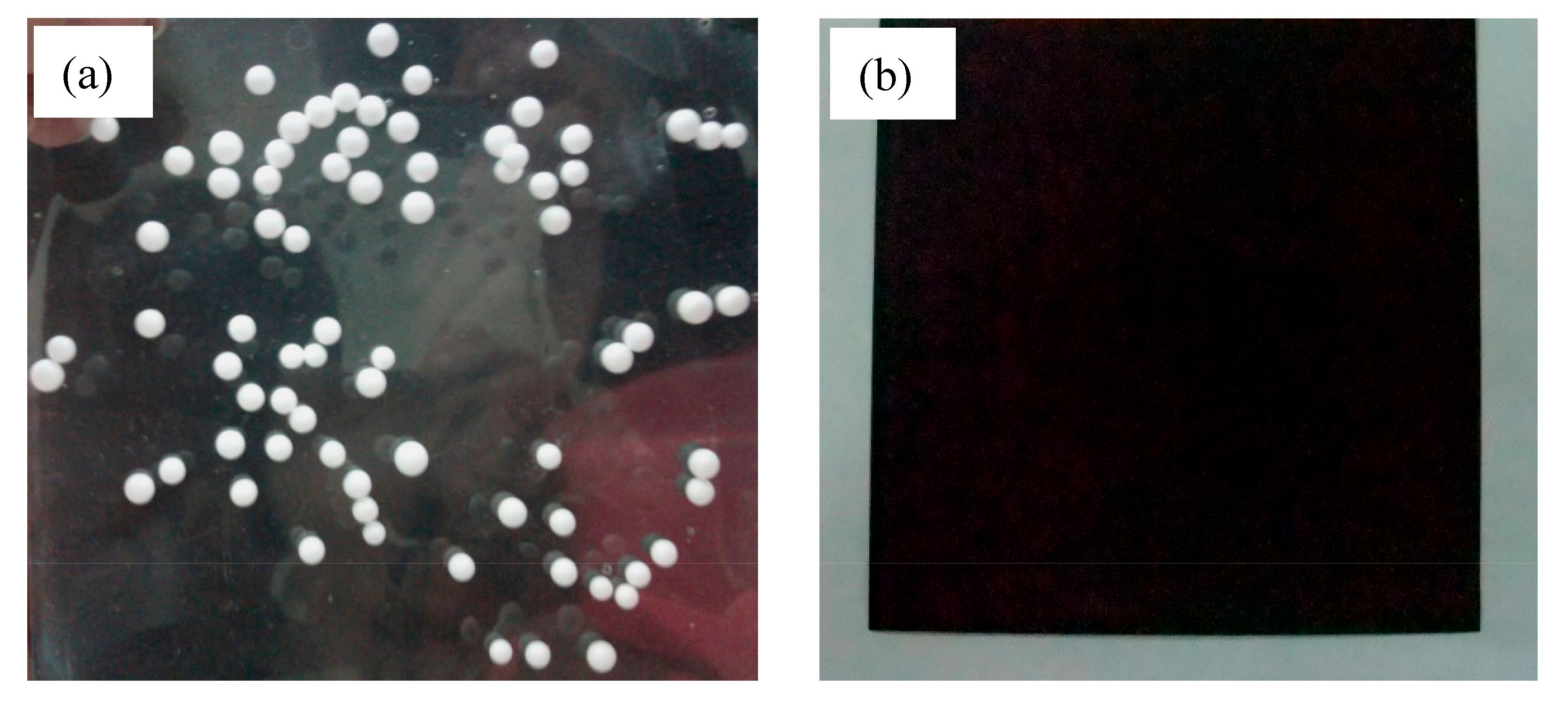
Antistatic tests: (**a**) pure WPU film, and (**b**) WPU/20wt% (PANDB/χ-Al_2_O_3_) conducting blend film.

The surface resistances of pure WPU film and WPU/20wt% (PANDB/χ-Al_2_O_3_) blend films are around 6.0 × 10^10^ and 1.5 × 10^4^ Ω/sq., respectively. It can be found that adding the 20wt% (PANDB/χ-Al_2_O_3_) conducting nanocomposite into the WPU matrice can significantly reduce the surface resistance of the WPU film from 6.0 × 10^10^ to 1.5 × 10^4^ Ω/sq. This is due to the formation of conducting paths through the WPU film by containing 20wt% conducting PANDB/χ-Al_2_O_3_ nanocomposite. Figure 6 shows an antistatic comparison between the pure WPU film (Figure 6a) and the WPU/20wt%(PANDB/χ-Al_2_O_3_) blend film (Figure 6b). The surfaces of the two films are first abraded and then placed among foamed polystyrene (PS) spheres. Clearly, many PS spheres are adsorbed on the pure WPU film, yet are not found on the WPU/20wt% (PANDB/χ-Al_2_O_3_) blend film. This phenomenon indicates that the WPU/20wt% (PANDB/χ-Al_2_O_3_) conducting blend film can function as a promising antistatic or electrostatic discharge material.

## 4. Conclusions

In this study, an in situ polymerization method was used to synthesize conducting PANDB/χ-Al_2_O_3_ core-shell nanocomposites. For the synthesized conducting core-shell nanocomposites, the core is a χ-Al_2_O_3_ nanoflake and the shell is conducting PANDB. The maximum electrical conductivity of the conducting PANDB/χ-Al_2_O_3_ core-shell nanocomposite is 1.7 × 10^−1^ S/cm when the AN/χ-Al_2_O_3_ weight ratio is higher than 1.5. The TEM and FE-SEM images show that the χ-Al_2_O_3_ nanoflakes have been thoroughly coated by PANDB to form a core-shell structure when the weight ratio of AN/χ-Al_2_O_3_ is higher than 1.5. Furthermore, the TEM images of the conducting PANDB/χ-Al_2_O_3_ core-shell nanocomposites indicate that the thickness of the PANDB layer can be increased as the weight ratio of AN/χ-Al_2_O_3_ is increased. It can be found that the optimum weight ratio of AN/χ-Al_2_O_3_ is identified as 1.5 in this study. It can be found that adding a 20wt% (PANDB/χ-Al_2_O_3_) conducting nanocomposite into the WPU matrix can significantly reduce the surface resistance of the WPU film from 6.0 × 10^10^ to 1.5 × 10^4^ Ω/sq. The result indicates that the WPU/20wt% (PANDB/χ-Al_2_O_3_) conducting blend film can function as a promising antistatic or electrostatic discharge material. In future work, the synthesized conducting PANDB/χ-Al_2_O_3_ core-shell nanocomposite can be used as a novel and multiple functional reinforcement and be blended with semicrystalline and glassy thermoplastics, elastomers, or epoxy resins. Thus, PANDB is expected to provide electrical conductivity, and χ-Al_2_O_3_ nanoflakes are expected to enhance the mechanical properties of the polymer matrix. Meanwhile, the synthesized product can be used as antistatic and static discharge material and shows good potential for use in chemical sensors, biosensors, and anticorrosion.

## Figures and Tables

**Table 1:** Influence of the weight ratio of AN/χ-Al_2_O_3_ on the electrical conductivity of the conducting PANDB/χ-Al_2_O_3_ core-shell nanocomposites.

Weight Ratio of AN/χ-Al_2_O_3_	Conductivity (S/cm)
Pure PANDB	6.3 × 10^−1^
0.5	0.6 × 10^−1^
1.0	1.1 × 10^−1^
1.5	1.7 × 10^−1^
2.0	1.7 × 10^−^^1^

## Data Availability

The data that support the findings of this study are available from the corresponding author upon reasonable request.
